# Genetic Diversity and Physiological Performance of Portuguese Wild Beet (*Beta vulgaris* spp. *maritima*) from Three Contrasting Habitats

**DOI:** 10.3389/fpls.2016.01293

**Published:** 2016-08-31

**Authors:** Isa C. Ribeiro, Carla Pinheiro, Carla M. Ribeiro, Maria M. Veloso, Maria C. Simoes-Costa, Isabel Evaristo, Octávio S. Paulo, Cândido P. Ricardo

**Affiliations:** ^1^Instituto de Tecnologia Química e Biológica, Universidade NOVA de LisboaOeiras, Portugal; ^2^Faculdade de Ciências e Tecnologia, Universidade NOVA de LisboaCaparica, Portugal; ^3^Computational Biology and Population Genomics Group, Centre for Ecology, Evolution and Environmental Changes, Faculdade de Ciências da Universidade de LisboaLisboa, Portugal; ^4^INIAV, Unidade de Investigação de Biotecnologia e Recursos GenéticosOeiras, Portugal; ^5^Instituto Superior de Agronomia, Linking Landscape, Environment, Agriculture and Food, Universidade de LisboaLisboa, Portugal; ^6^INIAV, Unidade de Investigação de Sistemas Agrários e Florestais e Sanidade VegetalOeiras, Portugal

**Keywords:** physiological characterization, population structure, biomass, crop wild relatives, allele richness

## Abstract

The establishment of stress resilient sugar beets (*Beta vulgaris* spp. *vulgaris*) is an important breeding goal since this cash crop is susceptible to drought and salinity. The genetic diversity in cultivated sugar beets is low and the beet wild relatives are useful genetic resources for tolerance traits. Three wild beet populations (*Beta vulgaris* spp. *maritima*) from contrasting environments, Vaiamonte (VMT, dry inland hill), Comporta (CMP, marsh) and Oeiras (OEI, coastland), and one commercial sugar beet (Isella variety, SB), are compared. At the genetic level, the use of six microsatellite allowed to detect a total of seventy six alleles. It was observed that CMP population has the highest value concerning the effective number of alleles and of expected heterozygosity. By contrast, sugar beet has the lowest values for all the parameters considered. *Loci* analysis with STRUCTURE allows defining three genetic clusters, the sea beet (OEI and CMP), the inland ruderal beet (VMT) and the sugar beet (SB). A screening test for progressive drought and salinity effects demonstrated that: all populations were able to recover from severe stress; drought impact was higher than that from salinity; the impact on biomass (total, shoot, root) was population specific. The distinct strategies were also visible at physiological level. We evaluated the physiological responses of the populations under drought and salt stress, namely at initial stress stages, late stress stages, and early stress recovery. Multivariate analysis showed that the physiological performance can be used to discriminate between genotypes, with a strong contribution of leaf temperature and leaf osmotic adjustment. However, the separation achieved and the groups formed are dependent on the stress type, stress intensity and duration. Each of the wild beet populations evaluated is very rich in genetic terms (allelic richness) and exhibited physiological plasticity, i.e., the capacity to physiologically adjust to changing environments. These characteristics emphasize the importance of the wild beet ecotypes for beet improvement programs. Two striking ecotypes are VMT, which is the best to cope with drought and salinity, and CMP which has the highest root to shoot ratio. These genotypes can supply breeding programs with distinct goals.

## Introduction

Sugar beet (*Beta vulgaris* spp. *vulgaris*) is a crop of great importance for the sugar industry, contributing with 20% to the world sugar production (FAO, 2009). High and stable production is a major priority, but this crop, usually rainfed, is very sensitive to dryer growing seasons, which may lead to up to 40% yield losses (Pidgeon et al., 2001). Climatic change is predicted to cause decrease in soil water availability and to reduce water quality for agriculture with major impact on sugar beet productivity. The anticipated scenario of water shortage can imply that the sugar beet production will become dependent on watering, with increased costs of production and the risk of soil salinization. To increase the competitiveness of sugar production from beet and decrease the dependence on sugar cane, focus should thus be put on the improvement of sugar beet yield.

Although, some variability is found within sugar beet cultivars (e.g., Ober and Luterbacher, 2002) it is considered that the crop lacks sufficient genetic variation to cope with stress. Indeed, it is admitted that sugar beet was selected from one single crop population (Francis, 2007), resulting in a very narrow genetic background and variability (Bartsch and Ellstrand, 1999; Fénart et al., 2008). Wild beet (*Beta vulgaris* spp. *maritima*), the ancestor of the existing beet crops, constitutes a valuable source of genetic variability for the beet group. These populations, spatially separated and with specific allele composition (ecotypes), could potentially carry stress tolerance traits. Wild beet was already used for sugar beet genetic improvement against pathogens (Panella and Lewellen, 2007). While its potential for improvement against abiotic factors has not yet been exploited, it has been proposed that the ability to accumulate compatible solutes is a breeding goal for abiotic stress tolerance (Bagatta et al., 2008; Ober and Rajabi, 2010; Wu et al., 2014), as well as the ability to accumulate Na^+^ and K^+^ (Abbasi et al., 2015). Europe is one of the most important centers for beet diversity (Maxted et al., 2008), the Iberian Peninsula being considered one center of origin of the *Beta* complex (OECD, 2001). A recent study of Andrello et al. (2015) using accessions from the whole distribution area of the *Beta* complex, including 44 accessions from Portugal, confirmed the existence of genetic diversity and of two distinct groups within ssp *maritima*, one Atlantic and another Mediterranean. In Portugal, there are wild beets populations in several distinct locations, characterized by a remarkable phenotypic variability (Frese et al., 1990; Monteiro et al., 2013). Some of these populations are adapted to very harsh conditions, such as salt marshes and seashore cliffs. Therefore, the Portuguese wild beet accessions are suitable for the screening of stress tolerant populations due to the selective pressure of the *Beta* populations (like drought and water salinization).

Molecular markers have been used in studies of plant populations providing insight in genetic structure and gene flow within wild populations (Manel et al., 2003; Fievet et al., 2007; Andrello et al., 2015). Microsatellites are highly polymorphic co-dominant markers widely dispersed throughout the eukaryotic genomes. The high content of genetic data yielded by microsatellites makes these markers one of the molecular tools of choice for population and biodiversity studies (Fénart et al., 2008; Smulders et al., 2010; Richards et al., 2014).

In this work we present the first characterization of Portuguese wild beet populations: Vaiamonte (dry inland, VMT), Oeiras (coastland, OEI) and Comporta (salt marsh, CMP), and include one sugar beet commercial variety (Isella) for comparison. We are interested in characterizing these wild beet populations regarding: (1) the genetic diversity; (2) the impact of drought and salt stress on biomass production and photosynthetic performance. We intend to identify genotypic specific responses to different environments and contribute with critical information useful for the design of sugar beet ideotype and sugar beet improvement.

## Materials and Methods

### Wild Beet Populations

Three Portuguese wild beet populations from distinct locations were used: two sea beets, one close to the seashore at Oeiras (OEI; 38°42′ N, 09°22′ W), the other on a Sado estuary salt marsh at Comporta (CMP; 38°23′ N, 08°47′ W); a third one, an inland ruderal beet, near Monforte (VMT; 39°07′ N, 07°29′ W). The germplasm was stored in *Banco Português de Germoplasma Vegetal, S. Pedro de Merelim* (Braga, Portugal) with the following accession numbers 4268 (CMP), 5252 (VMT) and 5253 (OEI). Information concerning those accessions can be found at http://eurisco.ecpgr.org.

Soil samples were collected at 30 cm depth and characterized according texture, pH, electric conductivity, main soluble cations and organic matter. **Table 1** summarizes the climate and soil characteristics of the sampling sites of the three beet populations.

**Table 1:** Location, climatic data, and soil characteristics for the three wild beet populations studied.

	Vaiamonte	Comporta	Oeiras
Location (GPS coordinates)	39°07′ N, 07°29′ W	38°23′ N, 08°47′ W	38°42′ N, 09°22′ W
Mean air temperature (°C)^∗^	15.2	16.2	16.4
Maximum temperature range (°C)^#^	12.5	13.5	9.7
Minimum temperature range (°C)^#^	5.4	9.4	5.7
n° months with negative temperature	5	6	0
Mean year rainfall (mm)^∗^	852	765	680
Soil texture classification	Sandy-loam	Silt-loam	Sandy-loam
Soil characteristics^∗∗^	
Coarse sand (%)	33.04 (±1.58) a	24.58 (±3.64) a	34.36 (±4.21) a
Fine sand (%)	44.44 (±1.23) a	8.26 (±0.54) b	36.18 (±4.38) c
Silt (%)	6.94 (±0.41) a	42.00 (±2.88) b	14.40 (±1.30) c
Clay (%)	15.60 (±0.70) a	25.12 (±0.95) b	15.08 (±1.13) a
Organic matter (g Kg^-1^)	35.15 (±2.81) a	84.70 (±8.53) b	20.28 (±2.54) c
pH^∗∗∗^	6.73 (±0.43) a	5.80 (±0.17) b	8.89 (±0.20) c
Electric conductivity (dS m^-1^)	0.50 (±0.08) a	1.30 (±0.33) b	0.47 (±0.03) a
Main soluble cations^∗∗∗∗^	
Ca (me L^-1^)	2.42 (±0.35) a,b	1.53 (±0.41) a	3.10 (±0.11) b
Mg (me L^-1^)	0.64 (±0.08) a	1.95 (±0.69) b	0.36 (±0.03) a
Na (me L^-1^)	0.59 (±0.07) a	5.65 (±1.31) a	0.79 (±0.20) a
K (me L^-1^)	0.79 (±0.29) a	0.76 (±0.17) b	0.27 (±0.10) a

*The climatic data refers to the 1971–2000 period (climatologic normal), available at www.ipma.pt/pt/oclima/normais.clima/1971-2000. The Soil analysis refers to the upper 30 cm of the topsoil. Duncan’s *post hoc* test after one-way ANOVA (*p* < 0.05) was performed in order to: compare treatments within each genotype (a, b, c). ^∗^Referring to the 1971–2000 period (climatologic normal). ^#^Calculated as the minimum (or maximum) monthly temperature range; Referring to the 1971–2000 period (climatologic normal). ^∗∗^Determined in five samples. ^∗∗∗^Determined in a 1:5 (wt/vol) aqueous soil slurry. ^∗∗∗∗^Determined in the soil water saturation extract.*

### Plants Growth Conditions and Sampling

For the biomass and physiological studies, seed glomerules were collected from the same plants used for genetic analysis. For comparison purposes we also used Isella, a commercial variety of sugar beet kindly provided by KWS SAAT AG seeds. Seed germination was performed as described (Felisberto-Rodrigues et al., 2010). Briefly, the glomerules were surface sterilized in 30% H_2_O_2_, scarified and germinated under sterile conditions in half-strength MS medium, at a constant temperature of 22°C until germination (which was defined as the number of days until radicle emergence). Since scarification was not necessary for sugar beet, seeds were imbibed in H_2_O and germinated in Petri dishes. After 2–3 days of germination, seedlings were transferred to one liter pots containing a mixture of coarse sand and peat (Shamrock). The experiments were conducted in a growth chamber, under 12 h photoperiod, 20–24°C, 60–70% relative humidity and photosynthetically active radiation (PAR) of circa 240 μmol m^-2^ s^-1^. Plants were watered every day with demineralized water to 80–90% of soil relative water content (SRWC, see definition in the next section). The stress treatments started when plants had 6–8 fully developed leaves (32–34 days from scarification or imbibition). One week before the beginning of the stress experiments all plants were watered with 200 mL of plant nutrient solution (Arnon, 1938; Arnon and Hoagland, 1940).

The drought stress was imposed by withholding water until the SRWC decreased to 13% (±1.2%), and the plants were then rewatered and allowed to recover for 1 day. The salinity treatments were induced by watering with 200 mM NaCl (10.3 dS m^-1^) or 500 mM NaCl solutions (13.8 dS m^-1^) while keeping SRWC at 80–90%. Plants were allowed to recover from salinity stress treatments by watering with excess demineralized water until the soil electroconductivity was reduced to lower than 0.7 dS m^-1^ (3 days).

Plants were physiologically evaluated (water status, osmotic adjustment, leaf gas-exchange parameters) at the beginning of the stress experiments (d0), and when the SRWC decreased to 68–57% (early drought), to 40–32% (intermediate stage), to 17–13% (severe/late drought) and after 1 day of stress recovery. The plants exposed to the salinity treatments were sampled at early, intermediate and late stress and salinity recovery taken into consideration soil electroconductivity and watering solution. For the 200 mM NaCl treatments: early stress (0.3 < soil Ece < 0.6 dS m^-1^); intermediate stress (0.7 < soil Ece < 1.5 dS m^-1^); late stress (1.5 < soil Ece < 2.3 dS m^-1^); recovery (soil Ece < 0.7 dS m^-1^). For the 500 mM NaCl treatment: early stress (0.9 < soil Ece < 1.7 dS m^-1^); intermediate stress 500 mM (1.4 < soil Ece < 2.9 dS m^-1^); late stress (2.6 < soil Ece < 4.3 dS m^-1^); recovery (soil Ece < 0.7 dS m^-1^). For biomass determination, plants were separated in shoot (all the aerial parts) and root and the biomasses determined after drying at 80°C.

### Monitoring of Soil Parameters

Soil relative water content, defined as [(pot weight – weight of the pot with totally dried peat)]/[(pot weight at field capacity – weight of the pot with totally dried peat)] × 100, was monitored daily. Pot evapotranspiration, defined as (pot weight on previous day – pot weight on the day of measurement)/(pot weight on day 0), was also monitored daily. Soil electroconductivity (ECs) was measured daily using a soil condutivimeter (Hanna Instruments, Inc., Woonsocket, RI, USA). Soil NaCl content was calculated using a calibration curve of electrical conductivity (EC) versus NaCl concentration. This curve was calculated using the ECs measured in pots saturated with NaCl solutions of increasing concentration (0–900 mM NaCl).

### Monitoring of Plant Water Relations and Leaf Gas Exchange

At the day of harvest the leaf water potential was taken at predawn (ψH_2_O), while the leaf (LRWC) and the root (RRWC) relative water contents were taken 4 h after the onset of illumination. Pre-dawn leaf water potential was measured with a Schölander pressure chamber (Model 1000, PMS Instruments, Co., Albany, NY, USA). For relative water content leaf disks and root slices were weighed to obtain fresh weight (FW), placed in Petri dishes containing water for 2 h, in the dark so they would become fully hydrated. Leaf disks and root slices were then re-weighed to obtain turgid weight (TW) and then dried at 80°C for 48 h to obtain dry weight (DW). RWC was calculated as: RWC = [(FW – DW)/(TW – DW)] × 100.

Leaf osmotic potential (ψs) was evaluated from samples collected at predawn. Leaf disks (8 mm, *n* = 5–6) were cut from the second youngest fully matured leaf during the pre-dawn period, frozen and kept at -80°C. The leaf osmotic potential was measured with HR-33T dew point microvoltimeter and the C-52 sample chambers (Wescor, Inc., Logan, UT, USA). Osmotic potential was corrected and the osmotic adjustment was calculated as described (Turner et al., 2007).

Leaf net photosynthetic rate and stomatal conductance measurements were taken every day within the period between 2 and 3 h after the beginning of illumination. Gas exchange was measured with a portable photosynthesis system (Li-6400, Li-Cor, Lincoln, NE, USA). Measurements were taken after reaching steady state level (monitored via CO_2_ and H_2_O graph functions) and took between 1 and 2 min to reach this level. Two to three measurements were made per plant on the most recently expanded leaf, and the leaf-chamber was maintained at 24°C and 43–45% relative humidity. The rate of molar air-flow inside the leaf chamber was 500 μmol mol^-1^. All measurements were taken at ambient CO_2_ concentration (370–400 μmol mol^-1^). The measurements were taken at a photosynthetic photon flux density (PPFD) of 240 μmol m^-2^ s^-1^, by using a red/blue light source (6400-02B LED) attached to the leaf chamber (6 cm^2^ area). Gas-exchange parameters were calculated automatically by the internal program of the Li-6400. Intrinsic water use efficiency was calculated as the ratio between the CO_2_ assimilation rate and the stomatal conductance.

### DNA Extraction, PCR Amplification, and Fragments Sizing

For the genetic studies, the native beet populations were field sampled according to Hawkes et al. (2000). Young leaves were collected from 30 plants of VMT and CMP and 34 plants of OEI wild beet populations. For sugar beet, six plants grown in the laboratory were sampled.

DNA was isolated from the beet young leaves using the DNeasy Plant Mini Kit (QIAGEN GmbH, Hilden Germany), according to the manufacturer’s protocol. DNA quantification was performed by spectrophotometry (NanoDrop 2000c, Thermo Scientific).

Twelve SSR *loci* which are evenly distributed over the *Beta* genome were tested: Bmb1, Bmb2, Bmb3, Bmb4, Bmb5, Bmb6 (Cureton et al., 2002); SB04, SB06, SB07, SB13, SB15 (Richards et al., 2004); BQ588629 (McGrath et al., 2007). These *loci* were selected on the basis of the publications and on personal information of Marion Nachtigall (JKI, Quedlinburg, Germany). The *loci* amplification was performed in a 10 μl solution using forward primers fluorescently labeled with WellRED dyes (D3 or D4) at the 5′-end and unlabeled reverse primers. PCR contained 1x reaction buffer, 2.3 mM MgCl_2_, 0.2 mM dNTPs, 2 pmol of each primer, 0.2 units of Taq polymerase (Pharmacia) and 20 ng of genomic DNA. The PCR was programmed as follows: 3 min at 94°C for the initial denaturation, followed by 30 cycles of denaturation at 94°C for 30 s, annealing at optimum Ta for 30 s and extension at 72°C for 1 min. A final extension step at 72°C for 7 min and the reaction was finished with a continuous cycle at 4°C. The reactions were conducted in a Biometra TGradient thermocycler. The PCR reactions were carried out separately for each microsatellite, and mixtures of PCR products of different markers with different dyes (or distinct allele size ranges) were prepared for simultaneous detection of the amplified alleles. Subsequently, 1.0 μl of the PCR mixture was added to 24 μl formamide and 0.5 μl fragment size standard labeled with WellRED dye D1. Capillary electrophoresis was performed to separate the PCR products using the CEQ 8000 Genetic Analysis System (Beckman Coulter). The size of the amplified bands was determined based on a standard internal size included with each sample. The precise allele sizes generated from each PCR was determined using the fragment analysis software of the CEQ8000.

### Data Analysis

On the R platform (version 2.15.1) we used: Mann–Whitney test for univariate statistical analysis (Boulesteix, 2009), Duncan’s *post hoc* test after one-way ANOVA (package agricolae); Ade4TkGUI for principal component analysis (PCA) and hierarchical clustering (Thioulouse and Dray, 2007); The Pearson’s product moment correlation coefficient was calculated using the cor.test in order to disclose significant relationships between principal components and the variables analyzed.

Microchecker software v2.2.3 (van Oosterhout et al., 2004) was used for the detection of null alleles, stuttering and allele dropout. Hardy–Weinberg equilibrium departures were tested for the three wild beet populations using the Fisher exact test (Genepop v3.4; Raymond and Rousset, 1995). The linkage disequilibrium was also tested with Genepop, where (F) is per *locus* and sample; Fstat was used to test whether there is a significant deficit or excess of heterozygotes and to calculate the allelic richness (Goudet, 2002). Genetic diversity was measured as the number of alleles per *locus* (Na), and observed and expected heterozygosities (Ho and He) using Genetix v4.05 (Belkhir et al., 2004). GenAlex6 program package (Peakall and Smouse, 2006) was used to assess the number of private alleles, to calculate the pair wise standard genetic distances (Nei, 1972) and the standard *F_ST_* (via Frequency) and the polymorphic information content (PIC). Population structure was assessed by the Bayesian model-based approach implemented in the STRUCTURE v.2.3 software (Pritchard et al., 2000).

## Results

### Microsatellite Genetic Diversity and Genetic Differentiation

The genetic diversity of the beet populations was studied through a microsatellite (SSR) genetic diversity analysis. Of the 12 microsatellite primer pairs initially selected to perform the study, three of them (Bmb2, Bmb3, Bmb5) failed to amplify some samples or yielded fragments of many sizes. For three other *loci* (Bmb1, Bmb4, Bmb6), the statistics analysis showed that the Hardy–Weinberg equilibrium of the populations was not met and, consequently, these *loci* were not used for further analysis. The *loci* SB04, SB06, SB07, SB13, SB15, and BQ588629 were polymorphic (**Table 2**). A total of seventy six alleles were scored in the wild beet populations (90 plants) and the sugar beet (six plants), the number of alleles ranging from three (SB13 *locus*) to 15 (SBO7 and BQ588629 *loci*). All the *loci* but one had PIC values higher than 0.70, thereby indicating that they can be useful diversity indicators. The *locus* SB07 displayed higher values for N_e_ and H_e_ with a PIC value of 0.897. Conversely, *locus* SB13 displayed the lowest PIC value (0.564), number of alleles and expected diversity (He; **Table 2**). Concerning allelic richness (Ar), significant differences were observed between the wild beet (ranging from 7.67 to 10.17) and the sugar beet (2.00). The CMP population displayed the highest mean values concerning the analyzed parameters (**Table 2**). Sugar beet has the lowest values for all these parameters. The number of private alleles (defined as those found in a single population) was also higher for CMP than for the other populations. Expected average heterozygosity across all populations per *locus* ranged from 0.539 (SB13) to 0.762 (SB07; **Table 2**).

**Table 2:** Number of individuals per population (N), number of alleles per locus (N_a_), effective number of alleles (N_e_), number of private alleles (Npa), allelic richness (A_r_), expected and observed heterozygosity (H_e_, H_o_), polymorphic information content (PIC) for the *loci* SB04, SB06, SB07, SB013, SB15, and BQ588629 on the studied beet populations (Pop).

		locus					
Population		SB04	SB06	SB07	SB13	SB15	BQ588629
OEI	N			34			
	N_a_	5	8	15	5	9	11
	N_e_	2.1	3.9	10.6	3.0	3.5	5.4
	Npa			0.83			
	Ar			9.17			
	H_e_	0.529	0.824	0.853	0.647	0.647	0.765
	H_e_	0.516	0.747	0.906	0.663	0.718	0.816
VMT	N			30			
	N_a_	6	6	10	3	10	10
	N_e_	4.3	3.5	4.3	1.9	6.3	5.0
	Npa			1.33			
	A_r_			7.67			
	H_e_	0.767	0.833	0.433	0.300	0.833	0.633
	H_e_	0.769	0.714	0.767	0.480	0.841	0.799
CMP	N	7	3	0	5	29	30
	N_a_		8	13		12	15
	N_e_	2.6	5.4	8.0	2.8	6.8	6.4
	Npa			1.50			
	A			10.17			
	H_e_	0.567	0.833	0.833	0.667	0.897	0.9
	H_e_	0.609	0.816	0.875	0.639	0.852	0.843
SB	N	2	2	2	6 2	2	2
	N_a_						
	N_e_	1.9	2.0	2.0	1.6	1.9	1.4
	Npa			0.00			
	A_r_			2.00			
	H_e_	0.833	1.000	1.000	0.500	0.833	0.333
	H_e_	0.486	0.500	0.500	0.375	0.486	0.278
All populations	PIC	0.710	0.752	0.897	.564	0.838	0.821
**Population**	**SB04**	**SB06**	**SB07**	**SB13**	**SB15**	**BQ588629**	**Mean**
F-statistics for all populations						
Mean H_e_	0.595	0.694	0.762	0.539	0.724	0.684
Mean H_o_	0.674	0.873	0.780	0.529	0.803	0.658
F_5T_	0.234	0.102	0.146	0.172	0.143	0.129	0.154
F-statistics for wild beet populations					
Mean H_e_	0.631	0.759	0.849	0.594	0.804	0.819
Mean H_o_	0.621	0.83	0.706	0.538	0.792	0.766
F_ST_	0.121	0.03	0.054	0.019	0.056	0.031	0.052

**FIGURE 1:**
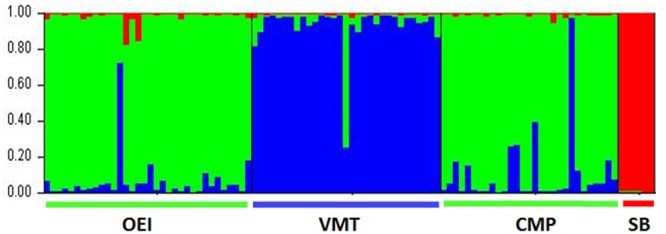
**Portuguese wild beets populations clustering according to SSR analysis using the package STRUCTURE and the *loci* SB04, SB06, SB07, SB013, SB15, and BQ588629.** Accessions are organized by region of provenance and each individual is represented by a vertical line segmented into K colored sections. The black vertical lines separate different regions. The best fitting model according to Evannno’s is *k* = 3.

The genetic differentiation among the populations, i.e., the proportion of the total genetic diversity that separates the populations, was evaluated using the F-statistics. When all the beets were considered, the F_ST_ value was 0.154 (**Table 2**), which indicates a moderate differentiation between the populations. In order to obtain information about structure in beet populations based on allele frequencies we used the STRUCTURE method. The computation of the Evanno’s K indicated *K* = 3 as the most likely number of genetic clusters. **Figure 1** show that wild see beets (OEI and CMP), inland ruderal beet (VMT) and sugar beet cultivar (SB) constituted different genetic clusters. The SB population is distinct from the wild beets and no distinction was detected between OEI and CMP while VMT is seen to be genetically distinct.

### Growth under Non-limiting Conditions

**FIGURE 2:**
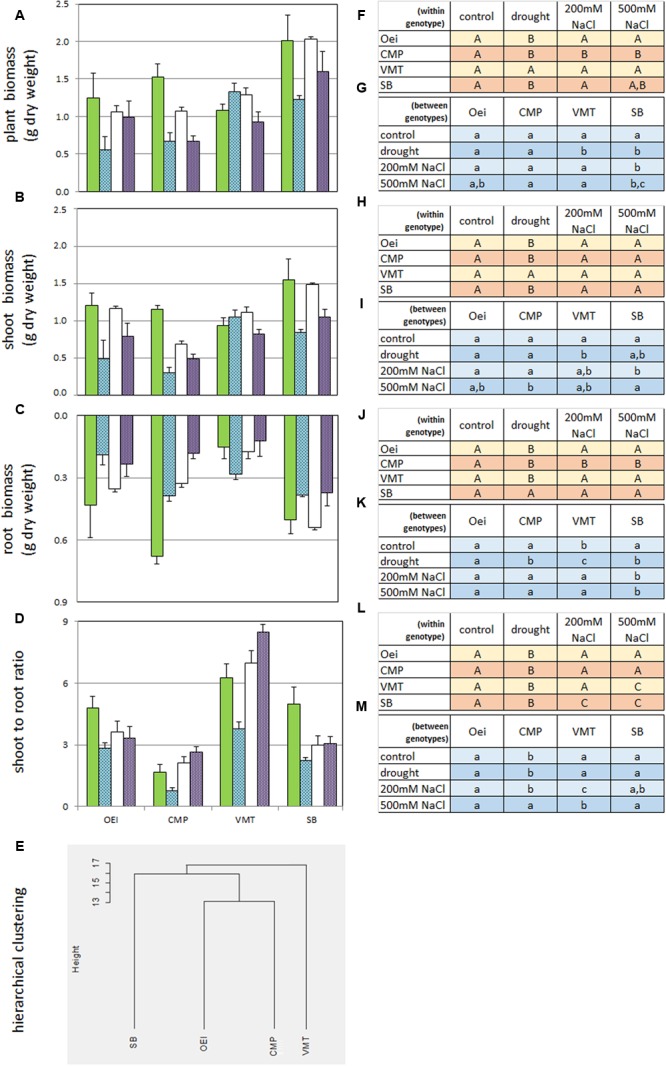
**Impact of the stress treatments on biomass production and partitioning on the wild beet populations (OEI – Oeiras; CMP – Comporta; VMT – Vaimonte) and sugar beet var. Isella (SB).** Values are the mean (+ standard error; *n* = 4–6) and represent the increase in g of dry weight per plant since the start of the treatments. **(A)** Plant biomass, **(B)** Shoot biomass; **(C)** Root biomass; **(D)** Shoot to root ratio; **(E)** Hierarchical clustering (Euclidean distance) of the several genotypes based on the biomass data **(A–D)**. **(F–M)** Duncan’s *post hoc* test after one-way ANOVA (*p* < 0.05). **(F,H,J,L)** Treatments within each genotype; **(G,I,K,M)** treatment between genotypes. Green bars – control; blue bars – prolonged drought; empty bars – prolonged salinity stress under 200 mM NaCl watering; violet bars – prolonged salinity stress under 500 mM NaCl watering.

The sugar beet and the three wild beets exhibited distinct biomass production and partitioning (**Figure 2**). At the end of the assay it was recorded a tendency for higher, but not significant, SB biomass.

It was found that genotypes differ in the water consumption and leaf-gas exchange properties (**Table 3**). Typically OEI and SB exhibited similar gas-exchange characteristics, except for the photosynthetic rate. The pot evapotranspiration rate indicates that the several genotypes had distinct water consumptions, SB and CMP being the ones with higher water requirements. Our data also show that SB while consuming more water, displayed the lower stomatal conductance and photosynthetic rate.

**Table 3:** Physiological characterization of the several wild beets and of the sugar beet at the onset of the stress assays (day 0).

	Photosynthetic rate (μ mol CO_2_ m^-2^ s^-1^)	Leaf stomatal conductance (mol H_2_O m^-2^s^-1^)	Internal CO_2_ concentration (μmol CO_2_ mol^-1^)	Leaf temperature (°C)	Leaf vapor pressure deficit, Vpdl (kPa)	Potevapotranspiration rate (ml H_2_O lost g^-1^ soil)
OEI	11.3 (±0.3) a	0.26 (±0.01) a	332 (±1) a	23.9 (±0.3) a	1.5 (±0.03) a	0.035 (±0.004) a
SB	9.2 (±0.3) b	0.19 (±0.03) a	290 (±26) a	23.6 (±0.1) a	1.5 (±0.06) a	0.052 (±0.004) b
CMP	11.4 (±0.3) a	0.34 (±0.03) b	383 (±5) b	22.6 (±0.1) b	1.1 (±0.03) b	0.047 (±0.003) b
VMT	9.9 (±0.3) c	0.45 (±0.04) c	325 (±1) a	22.7 (±0.1) b	0.9 (±0.04) c	0.033 (±0.003) a

*Duncan’s *post hoc* test after one-way ANOVA (*p* < 0.05) was performed in order to: compare treatments within each genotype (a, b, c). a, b, c, statistically significant differences according to the Duncan test (*p* < 0.05).*

### Biomass Production under Stress

As the impact of lower water availability (or lower water quality) on biomass production and partitioning is an important aspect of *Beta* genus as a crop, the effects of prolonged drought and salinity on the capacity of biomass production and partitioning of the several genotypes was tested (**Figure 2**). Drought and salinity effects were distinct and genotype dependent. VMT biomass production contrasted from all the other genotypes since it was stimulated by drought. It was also observed that, except for VMT, the impact of drought stress on biomass was more severe than the impact of salinity (**Figure 2**). The two salinity treatments were further distinguished since 500 mM NaCl, but not 200 mM NaCl, caused the decrease of biomass in all genotypes considered. Under the 200 mM NaCl regime, VMT biomass was not affected while CMP biomass was negatively affected (**Figure 2**). Despite the severity of the imposed stresses, all plants from all genotypes were able to recover. Taking into consideration **Figures 1** and **2E**, two sub-groups can be devised: one represented by VMT, the other represented by OEI and CMP. The effects of drought and salinity on biomass production lead to the working hypothesis that the three wild beets regulate differently carbon assimilation and water consumption under stress.

### Physiological Performance under Drought and Drought Recovery

The rate of water depletion from the soil was monitored daily, the water loss from the soil being gradual and slow (Supplementary Figure S1A). However, there were differences in the rate of soil water depletion between the beets, indicating distinct water requirements and/or distinct capacities for controlling water consumption. VMT, which was found to be less demanding, exhibited a different strategy under stress, spending water faster than the other genotypes. On the other hand, the genotypes more demanding in terms of water needs (CMP and SB) exhibited an intermediate consumption under stress, while OEI genotype demonstrated the better control of water spending.

**FIGURE 3:**
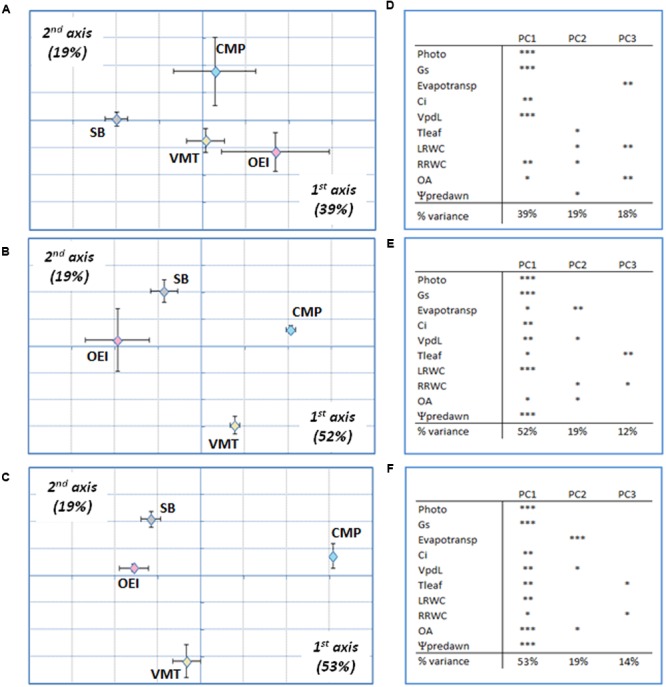
**Principal components analysis (PCA) of drought responses at early stress (**A,D**), late stress (**B,E**) and recovery (**C,F**) of the wild beets (OEI, CMP, VMT) and sugar beet to a progressive soil water deficit. (D–F)** Principal components loadings (% of variation explained) and Pearson’s product moment correlation coefficient for each variable (^∗^*p* < 0.05, ^∗∗^*p* < 0.01; ^∗∗∗^*p* < 0.001). Four biological replicas were considered per genotype and stress level (raw data available as Supplementary Table S1).

Principal component analysis followed by between groups analysis (BGA) was performed in order to get a global view of the drought effects on the physiology of the beet plants. It was possible to observe that the discrimination along the first, second, and third axis was driven by stress levels and intensities, not by genotypes (data not shown). In order to analyze specific responses at a similar drought intensity, early stress, severe stress and recovery were considered separately. This strategy allowed to verify that the physiological responses to drought from the different beets were distinct (**Figure 3**). Regarding the early stress responses (**Figures 3A,D**), SB was separated from the wild beets along the first component (39%), while CMP group distinctly from VMT and OEI along the second component (19%). The VMT population was separated from the remaining beets on the third component (18%), which was significantly correlated with the variables pot evapotranspiration, leaf RWC and OA. The physiological responses of the beets to severe drought (**Figures 3B,E**) showed also genotypic specific responses although with a distinct pattern. OEI and SB group together and were only separated along the third axis (12%), due to Tleaf and OA. CMP and VMT separates from OEI and SB along the first axis (52%) and were discriminated along the second axis (19%) due to the contribution of pot evapotranspiration, Vpdl, the root RWC and OA. Regarding plant recovery (**Figures 3C,F**), it was also observed that OEI and SB group together being separated only along the third axis (14%). But contrarily to severe drought stress, VMT groups with SB and OEI along the first axis (53%, with the significant contribution of all parameters considered, except the pot evapotranspiration). VMT was separated along the second component, due to OA, pot evapotranspiration and Vpdl (reflecting Tleaf). Taken together, our data have identified OA and water consumption as relevant parameters for VMT discrimination from the other genotypes under drought.

### Physiological Performance under Salt Stress and Salinity Recovery

The physiological performance under two distinct levels of salinity (watering with 200 mM NaCl and 500 mM NaCl) was monitored. The soil ECs profile shows a slow stress imposition rate for both salinity treatments (Supplementary Figure S1B). Similarly to what was observed for drought experiments, PCA followed by BGA revealed that discrimination along the first, second, and third axis was driven by stress levels and intensities, not by genotypes (data not shown).

**FIGURE 4:**
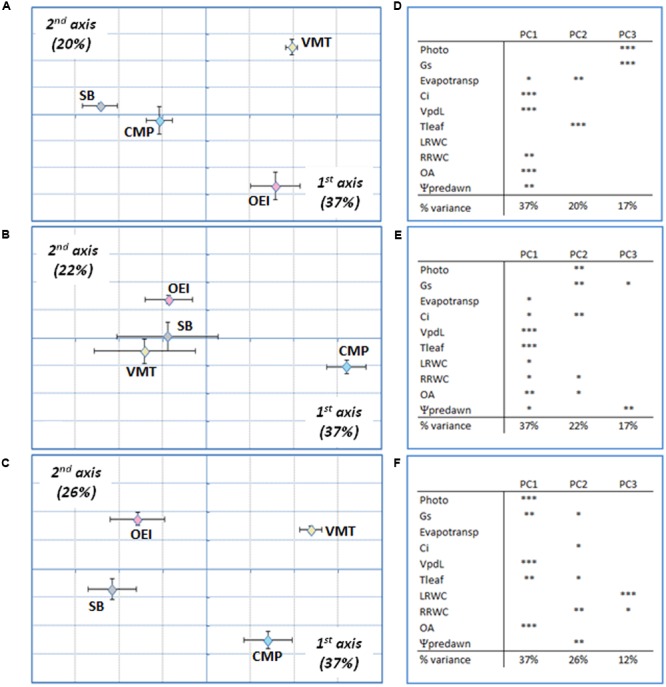
**Wild beets (OEI, CMP, VMT) and sugar beet responses submitted to a progressive salinity stress induced by watering with 200 mM analyzed by PCA at early stress (**A,D**), late stress (**B,E**) and recovery for 3 days (**C,F**). (A–C)** Plot showing the variance along the first two components; **(D–F)** Principal components loadings (% of variation explained) and Pearson’s product moment correlation coefficient for each variable (^∗^*p* < 0.05, ^∗∗^*p* < 0.01; ^∗∗∗^*p* < 0.001). Four biological replicas were considered per genotype and stress level (raw data available as Supplementary Table S1).

**FIGURE 5:**
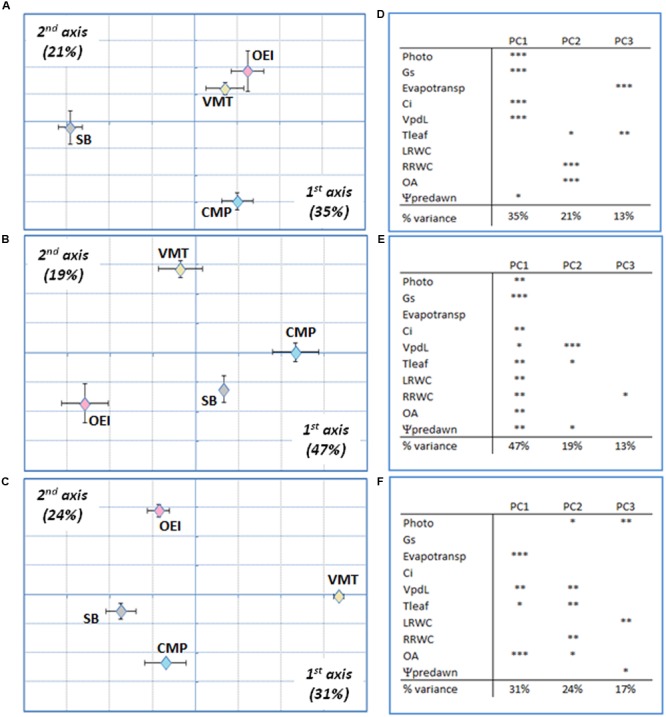
**Wild beets (OEI, CMP, VMT) and sugar beet responses submitted to a progressive salinity stress induced by watering with 500 mM NaCl analyzed by PCA at early stress (**A,D**), late stress (**B,E**) and recovery for 3 days (**C,F**). (D–F)** Principal components loadings (% of variation explained) and Pearson’s product moment correlation coefficient for each variable (^∗^*p* < 0.05, ^∗∗^*p* < 0.01; ^∗∗∗^*p* < 0.001). Four biological replicas were considered per genotype and stress level (raw data available as Supplementary Table S1).

A more detailed PCA and BGA analysis was performed by considering separately the two salinity treatments (**Figures 4** and **5**) and each stress level individually. Considering the 200 mM NaCl stress treatment (**Figure 4**), and at the early stages of stress, SB and CMP group together and the available data do not allowed to further discriminate them (**Figure 4A**). They were separated from VMT and OEI along the first axis (37%), which were further distinguished along the second axis (20%) due to the contribution of pot evapotranspiration and Tleaf. At this stage stomata conductance and photosynthetic activity were not strong contributors for genotype discrimination. As the stress progressed, the responses shown by OEI, SB, and VMT allowed them to cluster in the PCA, while CMP (salt marsh adapted) was separated in the first axis (37%, **Figure 4B**). The genotypes exhibited the highest discrimination upon recovery (**Figure 4C**) as they were fully separated along the first and second axis (37 and 21%, respectively). The data show that OA and Vpdl (which reflect Tleaf) are relevant parameters, contributing to the separation along the first axis irrespective of the stress level/duration. A distinct pattern was observed when analyzing the physiological responses to an early stress induced by 500 mM NaCl (**Figure 5A**). In this case, SB was separated from the wild beets along the first axis (35%). The wild beets were separated along the second axis (CMP from VMT and OEI, 21%) and third axis (VMT from OEI, 13%). Again pot evapotranspiration, Tleaf and OA are relevant in the VMT discrimination. The treatment with 500 mM for longer periods (late stress) allowed for a more clear separation of all the genotypes (first and second axis, 47 and 19%, respectively), which may reflect the existence of distinct mechanisms that are only revealed under long and severe stress. In this case, pot evapotranspiration is the only parameter not significantly associated with genotype discrimination in the first axis while Tleaf and Ψpd were important in the second axis. During stress recovery, VMT was clearly separated from the other genotypes along the first axis (**Figure 5C**, 31%). The variance along the first axis was due to the responses in pot evapotransporation, Tleaf and OA. Comparing the two salinity treatments it was observed that: early salinity stress allowed to separate VMT from the others when watered with 200 mM NaCl but not under the 500 mM regime; when genotypes are submitted to the salinity treatments for some more time, CMP outgroups from the others, which is more visible under the 200 mM treatment; under recovery, CMP and VMT were separated from the others, the distinctiveness of VMP being more clear for the recovery from the 500 mM treatment. Under recovery, the parameters OA and pot evapotranspiration strongly contributed to the separation of VMT from the other genotypes.

## Discussion

As the Portuguese wild beets are naturally exposed to drought and salinity we have hypothesized that these wild beets have stress tolerance/resistance traits. In this work, we present the first molecular and biochemical characterization of Portuguese wild beets through the comparison of four populations, two wild sea beets (OEI and CMP), one inland ruderal beet (VMT) and one sugar beet cultivar (SB). Our study indicates that the SSRs we have used allowed to define the genetic structure of the beet populations. It is interesting that a recent work (Abbasi et al., 2015) demonstrates furthermore, the association of SB07 and SB15 markers with yield related traits, SB15 also be associated with saline responses. The levels of allelic diversity and heterozygosity are higher in the wild beet populations than in the SB, as also observed by Fénart et al. (2008) when comparing French wild beets with sugar beet cultivars. The high level of allelic diversity in the wild populations is most probably due to their mating system, since beets are wind-pollinated plants (Bartsch et al., 2003). The low level of genetic diversity in SB (A_r_ and H_e_ values) is associated with domestication and breeding processes, was already referred not only for *Beta* but also for other plant species (Gepts, 1998; Fénart et al., 2008; Santalla et al., 2010). The genetic differentiation among wild populations is small (*F*_ST_ = 0.052; **Table 2**). The inclusion of SB in the analysis raises F_ST_ value to 0.154 due to the decrease of the average H_e_ in the whole group. On the other hand, it is possible to separate the inland ruderal beet (VMT) from the wild sea beets (OEI and CMP) by using the STRUCTURE program, which demonstrate the existence of diversity within the Portuguese wild beets. It has been suggested that ruderal beets originated from cultivar seed escape (Arnaud et al., 2009). This hypothesis is supported by Saccomani et al. (2009) for Italian ruderal beets that grouped more closely to cultivated sugar beet than to sea beets. However, and by contrast, the Portuguese wild beet populations are closer to each other than to sugar beet, what does not support the hypothesis of seed escape for the VMT origin. Furthermore, the Portuguese VMT beet cannot be viewed as a feral form since there has not been sugar beet cultivation nearby. So, the VMT beets are typical wild beets whose origin is presently unknown and is quite different from the other two wild beets analyzed (CMP; OEI). Andrello et al. (2015) in a recent study on the genetic diversity of the *Beta* complex indicates that Mediterranean and Atlantic accessions form two distinct spatial groups and that gene flow is expected to occur between coastal sites. Richards et al. (2014) also found a genetic structure and a gene flow in *Beta maritima* along the Atlantic coast of France. Since OEI and CMP cluster together and are both sea beets from the Atlantic coast, we may consider that an identical process occurred between them.

The distinctiveness of VMT in relation to CMP and OEI is also visible in morpho-physiological features. Biomass accumulation in organs of marketable value is of great importance due to its implications with agricultural performance. Regarding sugar beet, dry matter accumulation in the root is of crucial significance and can even control photosynthetic efficiency (Humphries and French, 1969). This crop is usually grown with adequate levels of water and mineral nutrients, but it has been observed that stress, in particular drought, greatly affects dry matter accumulation in the root (Choluj et al., 2004; Monti et al., 2006). Our data show that CMP shoot to root ratio under control conditions is smaller than the shoot to root ratio of SB and of the other wild beets. CMP (salt marsh habitat) invested more in root growth (lowest shoot/root ratio) while the inland VMT ecotype preferentially invested in leaf formation. The observed differences support the use of VMT for leaf producing beets and CMP for root producing beets as the differences reflect genetic distinctiveness. These trends are also detected when the genotypes are submitted to prolonged drought and salinity, the genotypes displaying distinct physiological responses to stress. Our data show that VMT outgroups from the others. In sharp contrast with the remaining beets studied, VMT invested in both shoot and root production under drought (the investment in root biomass being higher than in shoot biomass). Under salinity, VMT exhibits the largest shoot to root ratio while keeping total biomass production, confirming that this genotype can be a useful source of genes. The ecological and functional significance of different root strategies needs to be determined but it is considered as a mechanism of adaptation to the soil and climatic resources (Saccomani et al., 2009).

Considering our study, Tleaf (and the temperature related Vpdl) and internal CO_2_ concentration typically discriminate genotypes. These parameters reflect differences in temperature control, higher Tleaf resulting from stomata closure and therefore reduced availability of CO_2_ at cellular level. In addition, leaf Ψpd and leaf OA discriminated between the genotypes. Our data relates well with those of Ober et al. (2005) that found genotypic variability regarding OA and stomatal conductance in sugar beet. Ober et al. (2005) and Tsialtas and Maslaris (2012) do not corroborate the hypothesis that crop yield improvements in *Beta* could be derived from selection for higher photosynthesis as indicated by Long et al. (2006). This could be related with the absence of a direct relationship between stomata closure and photosynthetic rate. Several authors working with sugar beet under drought or salinity have shown that photosynthetic activity decreases even if internal CO_2_ concentration remains relatively constant (Delfine et al., 2003; Bloch et al., 2006; Monti et al., 2006, 2007; Dadkhah and Moghtader, 2008; Daoud et al., 2008; Dadkhah, 2011; Tsialtas and Maslaris, 2012). According to Monti et al. (2006), CO_2_ concentration at carboxylation sites rather than CO_2_ internal concentration is more affected. The change in CO_2_ diffusion is related with Tleaf as CO_2_ diffusion decreases with temperature (in opposition to O_2_). Therefore, Tleaf regulation has a strong impact on CO_2_ assimilation. VMT shows the least increase in Tleaf, which is accompanied by a distinct strategy in leaf osmotic adjustment and water potential at predawn, and controls intracellular water in a distinct way to CMP (and OEI). CMP is the genotype performing higher osmotic adjustments. However, in terms of biomass production it is not the best performer, which allows to postulate that osmotic adjustment is not the most predominant feature for stable biomass production under stress. Our data point out Tleaf as a useful parameter for germplasm screening. Tleaf regulation can reflect the adaptations to the environment and the geographical distribution of the Portuguese wild beets. These populations are originated from different environments, facing distinct temperature ranges and precipitation (**Table 1**). Andrello et al. (2015) found that the annual mean temperature is a relevant parameter for beet discrimination, and a possible motor for evolution. These authors describe a genetic diversification from South-East to North-West within sea beet accessions. VMT an inland genotype copes well with drought and salinity outstanding the sea beet CMP, which is an ecotype that grows in marshland.

The Portuguese wild beets CMP and VMT thus represent a valuable resource: CMP for root production under favorable conditions or when irrigation is an option; VMT for the genetic screening of abiotic stress resilience and biomass production under stress. To fully exploit their potential CMP and VMT should be evaluated under field conditions and in relation to the ideotype. Considering the market value of sugar beet, the ideotype should have a high root biomass and be able to attract a high proportion of assimilated carbohydrate in order to increase sugar yield. These two traits require alterations in partitioning and larger root cells. Smaller sugar beet roots are due to small cell size, not smaller cell number (Connor et al., 2011). Root anatomical characteristics influence sugar yield, the ability to compete for photoassimilate being related with the number of vascular rings in the root (Maiti et al., 2012). The selection of a sugar beet ideotype aims improving nutrient capturing capacity (Saccomani et al., 2009) and, so, should also consider the root vigor. This characteristic was found to be significantly associated with sugar beet yield (Biscarini et al., 2014) as higher root elongation rate is important to cope with stresses that limits the ability to absorb water.

## Conclusion

The Portuguese wild beets from different habitats activate distinct features when submitted to abiotic stress, what must be related to their genetic structure. Our data highlight VMT as a particularly interesting genotype for breeding programs as it is the less stress affected genotype. Drought is a major constraint for sugar beet production and VMT shows a stable biomass production under drought. Although under non-limiting conditions it invests in leaves, under drought it invests in root biomass. The ecotype that invest more in roots under non-limiting conditions is CMP, what is a desirable trend for sugar beet crops. The Portuguese wild beets can thus be considered relevant genetic reservoirs for sugar beet improvement, and the evaluation of field performance are the next steps that should be undertaken. A comparative study with other wild beets is needed in order to identify the molecular traits of interest when considering the selection of sugar beet ideotype.

## Author Contributions

CP, MV, and CPR designed the project; MV and CPR collected the plant material (seeds and leaves) and the soil samples for analysis; IR and CP performed the experimental stress assays under controlled conditions; CMR, MS-C, IE, and MV performed the molecular work; OP and MV conceived and designed the statistical analysis concerning the SSRs; IR, CP, MV, and CPR performed the analysis of the data and contributed to the writing; all the authors reviewed the manuscript.

## Conflict of Interest Statement

The authors declare that the research was conducted in the absence of any commercial or financial relationships that could be construed as a potential conflict of interest.

## Abbreviations

Ci: internal CO_2_ concentration
MS: Murashige and Skoog medium
Tleaf: leaf temperature

## References

[B1] Abbasi Z. Majidi M. M. Arzani A. Rajabi A. Mashayekhi P. Bocianowski J. (2015). Association of SSR markers and morpho-physiological traits associated with salinity tolerance in sugar beet (*Beta vulgaris* L.). *Euphytica* 205 785–797. 10.1007/s10681-015-1408-1

[B2] Andrello M. Henry K. Devaux P. Desprez B. Manel S. (2015). Taxonomic, spatial and adaptive genetic variation of *Beta* section *Beta*. *TAG. Theor. Appl. Genet.* 129 257–271. 10.1007/s00122-015-2625-7 26526552

[B3] Arnaud J.-F. Fénart S. Godé C. Deledicque S. Touzet P. Cuguen J. (2009). Fine-scale geographical structure of genetic diversity in inland wild beet populations. *Mol. Ecol.* 18 3201–3215. 10.1111/j.1365-294X.2009.04279.x 19627487

[B4] Arnon D. I. (1938). Microelements in culture-solution experiments with higher plants. *Am. J. Bot.* 25 322–325. 10.2307/2436754

[B5] Arnon D. I. Hoagland D. R. (1940). Crop production in artificial culture solutions and in soils with special reference to factors influencing yields and absorption of inorganic mutrients. *Soil Sci.* 50 463–484.

[B6] Bagatta M. Pacifico D. Mandolino G. (2008). Evaluation of the osmotic adjustment response within the genus *Beta*. *J. Sugar Beet Res.* 45 119–133. 10.5274/jsbr.45.3.119

[B7] Bartsch D. Cuguen J. Biancardi E. Sweet J. (2003). Environmental implications of gene flow from sugar beet to wild beet–current status and future research needs. *Environ. Biosaf. Res.* 2 105–115. 10.1051/ebr:2003006 15612276

[B8] Bartsch D. Ellstrand N. C. (1999). Genetic evidence for the origin of Californian wild beets (genus *Beta*). *Theor. Appl. Genet.* 99 1120–1130. 10.1007/s001220051316

[B9] Belkhir K. Borsa P. Chikhi N. Bonhomme F. (2004). *GENETIX 4.05*, logiciel sous window TM pour la genetique des populations. *Laboratoire Génome, Populations, Interactions, CNRS UMR 5000.* Montpellier: Université de Montpellier II.

[B10] Biscarini F. Stevanato P. Broccanello C. Stella A. Saccomani M. (2014). Genome-enabled predictions for binomial traits in sugar beet populations. *BMC Genet.* 15:87 10.1186/1471-2156-15-87 PMC4113669 25053450

[B11] Bloch D. Hoffmann C. M. Märländer B. (2006). Impact of water supply on photosynthesis, water use and carbon isotope discrimination of sugar beet genotypes. *Eur. J. Agron.* 24 218–225. 10.1016/j.eja.2005.08.004

[B12] Boulesteix A.-L. (2009). *WilcoxCV: Wilcoxon-Based Variable Selection in Cross-Validation. R Package Version 1.0-2.* Available at: https://cran.r-project.org/web/packages/WilcoxCV/WilcoxCV.pdf

[B13] Choluj D. Karwowska R. Jasińska M. Haber G. (2004). Growth and dry matter partitioning in sugar beet plants (*Beta vulgaris* L.) under moderate drought. *Plant Soil Environ.* 50 265–272.

[B14] Connor D. J. Loomis R. S. Cassman K. G. (2011). *Crop Ecology: Productivity and Management in Agricultural Systems* 2nd Edn. Cambridge: Cambridge University Press.

[B15] Cureton A. N. Burns M. J. Ford-Lloyd B. V. Newbury H. J. (2002). Development of simple sequence repeat (SSR) markers for the assessment of gene flow between sea beet (*Beta vulgaris* ssp maritima) populations. *Mol. Ecol. Notes* 2 402–403. 10.1046/j.1471-8286.2002.00253.x

[B16] Dadkhah A. Moghtader S. H. (2008). “Growth and gas exchange response of sugar beet (*Beta vulgaris* L.) cultivars grown under salt stress,” in *Photosynthesis. Energy from the Sun. 14th International Congress on Photosynthesis* eds Allen J. F. Gantt E. Golbeck J. H. Osmond B. (Dordrecht: Springer Netherlands) 1431–1434.

[B17] Dadkhah A. R. (2011). Effect of salinity on growth and leaf photosynthesis of two sugar beet (*Beta vulgaris* L.) cultivars. *J. Agric. Sci. Technol.* 13 1001–1012.

[B18] Daoud S. Harrouni C. Huchzermeyer B. Koyro H.-W. (2008). “Comparison of salinity tolerance of two related subspecies of *Beta vulgaris*: the sea beet (*Beta vulgaris* ssp. *maritima)* and the sugar beet *(Beta vulgaris ssp. vulgaris)*,” in *Biosaline Agriculture and High Salinity Tolerance* eds Abdelly C. Öztürk M. Ashraf M. Grignon C. (Berlin: Springer Science & Business Media) 115–129.

[B19] Delfine S. Tognetti R. Alvino A. Loreto F. (2003). Field-grown chard (*Beta vulgaris* L.) under soil water stress conditions: effect on antioxidant content. *Acta Hortic.* 618 329–335. 10.17660/ActaHortic.2003.618.38

[B20] FAO (2009). *Sugar Beet White Sugar. Agribusiness Hanbook.* Rome: European Bank and FAO.

[B21] Felisberto-Rodrigues C. Ribeiro I. C. Veloso M. Pinto Ricardo C. Pinheiro C. (2010). Germination under aseptic conditions of different ecotypes of wild beet (*Beta vulgaris* L. *ssp maritima).* *Seed Sci. Technol.* 38 517–521. 10.15258/sst.2010.38.2.24

[B22] Fénart S. Arnaud J.-F. De Cauwer I. Cuguen J. (2008). Nuclear and cytoplasmic genetic diversity in weed beet and sugar beet accessions compared to wild relatives: new insights into the genetic relationships within the *Beta vulgaris* complex species. *Theor. Appl. Genet.* 116 1063–1077. 10.1007/s00122-008-0735-1 18335202

[B23] Fievet V. Touzet P. Arnaud J.-F. Cuguen J. (2007). Spatial analysis of nuclear and cytoplasmic DNA diversity in wild sea beet (*Beta vulgaris* ssp. maritima) populations: do marine currents shape the genetic structure? *Mol. Ecol.* 16 1847–1864. 10.1111/j.1365-294X.2006.03208.x 17444897

[B24] Francis S. A. (2007). *Development of Sugar Beet.* Hoboken,NJ: Blackwell Publishing Ltd.

[B25] Frese L. Meijer E. Letschert J. (1990). New beet genetic resources from Portugal and Spain. *Zuckerindustrie* 115 950–956.

[B26] Gepts P. (1998). Origin and evolution of common bean:past events and recent trends. *HortScience* 33 1124–1130.

[B27] Goudet J. (2002). *Fstat, A Program to Estimate and Test Gene Diversities and Fixation Indices (Version 2.9.3.2).* Available at: http://www2.unil.ch/popgen/softwares/fstat.htm

[B28] Hawkes J. G. Maxted N. Ford-Lloyd B. V. (2000). *The ex situ conservation of plant genetic resources.* London: Kluwer Academic Publishers.

[B29] Humphries E. C. French S. A. (1969). Photosynthesis in sugar beet depends on root growth. *Planta* 88 87–90. 10.1007/BF00396118 24504840

[B30] Long S. P. Zhu X.-G. Naidu S. L. Ort D. R. (2006). Can improvement in photosynthesis increase crop yields? *Plant Cell Environ.* 29 315–330. 10.1111/j.1365-3040.2005.01493.x 17080588

[B31] Maiti R. Satya P. Rajkumar D. Ramaswamy A. (2012). *Crop Plant Anatomy.* Wallingford: CAB International.

[B32] Manel S. Schwartz M. K. Luikart G. Taberlet P. (2003). Landscape genetics: combining landscape ecology and population genetics. *Trends Ecol. Evol.* 18 189–197. 10.1016/S0169-5347(03)00008-9

[B33] Maxted N. Ford-Lloyd B. V. Kell S. P. (2008). “Crop wild relatives: establishing the context,” in *Crop Wild Relative Conservation and Use* eds Maxted N. Ford-Lloyd B. V. Kell S. P. Iriondo J. Dulloo E. Turok J. (Wallingford: CABI Publishing) 3–30.

[B34] McGrath J. M. Trebbi D. Fenwick A. Panella L. Schulz B. Laurent V. (2007). An open-source first-generation molecular genetic map from a sugarbeet x table beet cross and its extension to physical mapping. *Crop Sci.* 47 27–44. 10.2135/cropsci2006-05-0339tpg

[B35] Monteiro F. Romeiras M. M. Batista D. Duarte M. C. (2013). Biodiversity assessment of sugar beet species and its wild relatives: linking ecological data with new genetic approaches. *Am. J. Plant Sci.* 4 21–34. 10.4236/ajps.2013.48A003

[B36] Monti A. Barbanti L. Venturi G. (2007). Photosynthesis on individual leaves of sugar beet (*Beta vulgaris*) during the ontogeny at variable water regimes. *Ann. Appl. Biol.* 151 155–165. 10.1111/j.1744-7348.2007.00162.x

[B37] Monti A. Brugnoli E. Scartazza A. Amaducci M. T. (2006). The effect of transient and continuous drought on yield, photosynthesis and carbon isotope discrimination in sugar beet (*Beta vulgaris* L.). *J. Exp. Bot.* 57 1253–1262. 10.1093/jxb/erj091 16467409

[B38] Nei M. (1972). Genetic distance between populations. *Am. Nat.* 106 283–292. 10.1086/282771

[B39] Ober E. S. Le Bloa M. Clark C. J. A. Royal A. Jaggard K. W. Pidgeon J. D. (2005). Evaluation of physiological traits as indirect selection criteria for drought tolerance in sugar beet. *Field Crop Res.* 91 231–249. 10.1016/j.fcr.2004.07.012

[B40] Ober E. S. Luterbacher M. C. (2002). Genotypic variation for drought tolerance in *Beta vulgaris*. *Ann. Bot.* 89 917–924. 10.1093/aob/mcf093 12102517 PMC4233805

[B41] Ober E. S. Rajabi A. (2010). Abiotic stress in sugar beet. *Sugar Tech.* 12 294–298.

[B42] OECD (2001). *Consensus Document on the Biology of Beta vulgaris L. (Sugar Beet). OECD Environment, Health and Safety Publications. Series on Harmonization of Regulatory Oversight in Biotechnology, No 18.* Available at: www.oecd.org/ehs/

[B43] Panella L. Lewellen R. T. (2007). Broadening the genetic base of sugar beet: introgression from wild relatives. *Euphytica* 154 383–400. 10.1007/s10681-006-9209-1

[B44] Peakall R. Smouse P. E. (2006). GENALEX 6: genetic analysis in Excel. Population genetic software for teaching and research. *Mol. Ecol. Notes* 6 288–295. 10.1093/bioinformatics/bts460 PMC3463245 22820204

[B45] Pidgeon J. D. Werker A. R. Jaggard K. W. Richter G. M. Lister D. H. Jones P. D. (2001). Climatic impact on the productivity of sugar beet in Europe, 1961-1995. *Agric. For. Meteorol.* 109 27–37. 10.1016/S0168-1923(01)00254-4

[B46] Pritchard J. K. Stephens M. Donnelly P. (2000). Inference of population structure using multilocus genotype data. *Genetics* 155 945–959. 10835412 10.1093/genetics/155.2.945 PMC1461096

[B47] Raymond M. Rousset F. (1995). GENEPOP (version-1.2) – Population-genetics software for exact tests and ecumenicism. *J. Hered.* 86 248–249.

[B48] Richards C. M. Brownson M. Mitchell S. Kresovich S. Panella L. (2004). Length polymorphisms of simple sequence repeats in *Beta vulgaris*. *Mol. Ecol. Notes* 4 243–245.

[B49] Richards C. M. Reeves P. A. Fenwick A. L. Panella L. (2014). Genetic structure and gene flow in *Beta vulgaris* subspecies maritima along the Atlantic coast of France. *Genet. Resour. Crop Evol.* 61 651–662. 10.1007/s10722-013-0066-1

[B50] Saccomani M. Stevanato P. Trebbi D. McGrath J. M. Biancardi E. (2009). Molecular and morpho-physiological characterization of sea, ruderal and cultivated beets. *Euphytica* 169 19–29. 10.1007/s10681-009-9888-5

[B51] Santalla M. De Ron A. M. De La Fuente M. (2010). Integration of genome and phenotypic scanning gives evidence of genetic structure in Mesoamerican common bean (*Phaseolus vulgaris* L.) landraces from the southwest of Europe. *TAG. Theor. Appl. Genet.* 120 1635–1651. 10.1007/s00122-010-1282-0 20143041

[B52] Smulders M. J. Esselink G. D. Everaert I. de Riek J. Vosman B. (2010). Characterisation of sugar beet (*Beta vulgaris* L. *ssp. vulgaris)* varieties using microsatellite markers. *BMC Genet.* 11:41 10.1186/1471-2156-11-41 PMC2890681 20482800

[B53] Thioulouse J. Dray S. (2007). Interactive Multivariate Data Analysis in R with the ade4 and ade4TkGUI Packages. *J. Stat. Softw.* 22 1–14. 10.18637/jss.v022.i05

[B54] Tsialtas J. T. Maslaris N. (2012). Leaf physiological traits and its relation with sugar beet cultivar success in two contrasting environments. *Int. J. Plant Prod.* 6 15–36.

[B55] Turner N. C. Abbo S. Berger J. D. Chaturvedi S. K. French R. J. Ludwig C. (2007). Osmotic adjustment in chickpea (*Cicer arietinum* L.) results in no yield benefit under terminal drought. *J. Exp. Bot.* 58 187–194. 10.1093/jxb/erl192 17088363

[B56] van Oosterhout C. Hutchinson W. F. Wills D. P. M. Shipley P. (2004). micro-checker: software for identifying and correcting genotyping errors in microsatellite data. *Mol. Ecol. Notes* 4 535–538. 10.1111/j.1471-8286.2004.00684.x

[B57] Wu G. Q. Wang C. M. Su Y. Y. Zhang J. J. Feng R. J. Liang N. (2014). Assessment of drought tolerance in seedlings of sugar beet (*Beta vulgaris* L.) cultivars using inorganic and organic solutes accumulation criteria. *Soil Sci. Plant Nutr.* 60 565–576. 10.1080/00380768.2014.921579

