# Supplementary figures and images for: Genetic Diversity and Physiological Performance of Portuguese Wild Beet (Beta vulgaris spp. maritima) from Three Contrasting Habitats
Source: Front Plant Sci. 2016 Aug 31;7:1293. doi: 10.3389/fpls.2016.01293 (PMC5006101; doi:10.3389/fpls.2016.01293)

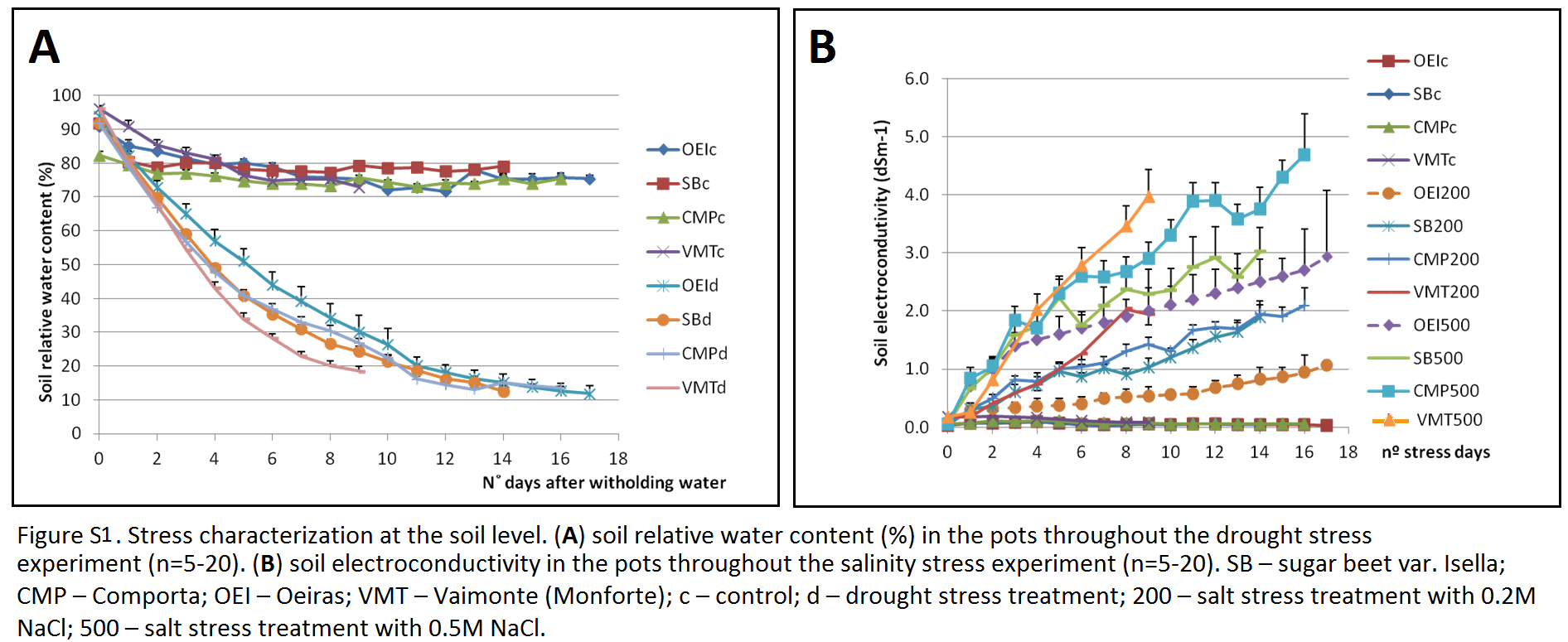

Supplement: Supplementary file 2 [file Image_1.TIF]
